# Indirect Self-Destructiveness in Women who Experience Domestic Violence

**DOI:** 10.1007/s11126-017-9560-5

**Published:** 2018-01-02

**Authors:** Konstantinos Tsirigotis, Joanna Łuczak

**Affiliations:** Department of Psychology, The Jan Kochanowski University in Kielce, Piotrków Trybunalski Branch, Słowackiego 114/118 str., 97-300 Piotrków Trybunalski, Poland

**Keywords:** Domestic violence, Women, Indirect self-destructiveness

## Abstract

Lives of people experiencing domestic or/and intimate partner violence abound in many unpleasant events and physical and psychological suffering, which affects their psychosocial functioning. The aim of this study was to explore indirect self-destructiveness as a generalised behavioural tendency and its manifestations in women experiencing domestic violence. The “Chronic Self-Destructiveness Scale” (CS-DS) was used to study two groups of women: 52 women aged 30–65 years (mean age: 40.15) using assistance of the Crisis Intervention Centre due to experienced domestic violence (V group) and 150 well-matched women not experiencing domestic violence (NV group). Women suffering domestic violence (V) obtained significantly higher scores than women not experiencing domestic violence (NV) for both the general index and a majority of CS-DS subscales; it was only for the A1 (Transgression and Risk) subscale that they achieved somewhat lower scores. Correlation coefficients between particular CS-DS subscales in the V group were higher than in the NV group; there were also certain differences in coefficients between the groups. Subscale factor analysis results were different too: only one factor was isolated in the V group while two were distinguished in the NV group. It can be inferred from the results that the intensity of indirect self-destructiveness as a generalised behavioural tendency as well as of most its categories was higher in women experiencing domestic violence. Tendencies and categories of indirectly self-destructive behaviours in women suffering domestic violence were more closely connected with one another, and the internal coherence of indirect self-destructiveness in those women might also be higher.

## Introduction

Lives of people experiencing domestic or/and intimate partner violence abound in many unpleasant events and physical and psychological suffering [cf. 1–4]. Violence in the family may concern all its members; it can also be of the mutual nature. In the case of physical violence, however, perpetrators tend to be men [5]. Although literature mentions psychological, physical, sexual and other forms of violence, they all actually come down to the psychological one: firstly, as soon as any other type of violence takes place, it automatically also becomes the psychological one; secondly, consequences of every violence type are almost always psychological too.

Domestic violence (DV) is defined as male aggression toward a female partner [6]. Domestic violence against women can be defined as any act or omission which, based on gender, causes death, physical, sexual or psychological injury and moral damage to women; it can be inflicted by individuals with or without family ties who are either related by natural bonds, affinity or express will, including sporadic relationships [7]. Due to the significance of the problem, it has also gained the attention of international organisations. Domestic violence is understood as all acts of physical, sexual, psychological or economic violence that occur within the family or domestic unit or between former or current spouses or partners, whether or not the perpetrator shares or has shared the same residence with the victim [8]. Proponents of that point of view draw attention to the fact that it is not the current place of residence of the perpetrator or an ongoing relationship with the victim that are the most important. The United Nations Declaration on the Elimination of Violence Against Women [9] defines violence against women taking place in the family in the following way: “Physical, sexual and psychological violence occurring in the family, including battering, sexual abuse of female children in the household, dowry-related violence, marital rape, female genital mutilation and other traditional practices harmful to women, non-spousal violence and violence related to exploitation”. A similar phenomenon/term is the battering relationship defined as the repeated use of physical, sexual or verbal force by someone against his intimate partner [10].

Behaviours causing harm to the individual, regardless of the intention, aim and degree of awareness of their negative consequences and irrespective of the time perspective (i.e. harm immediately vs. harm later) and object of harm (physical or psychological existence of the individual), can be referred to as self-destructive behaviours. A majority of authors most commonly understand the term “self-destructive behaviours” as behaviours belonging to the direct or acute self-destructiveness category, i.e. self-injuring, self-mutilation, suicide attempts and committed suicides. A distinction exists between direct and indirect threat and/or harm. The subject of this work is indirect self-destructiveness. The category is important because it almost imperceptibly generates unwished-for and harmful effects, though a great number of such behaviours are counted by most people among normal ones. Research on indirect or chronic self-destructiveness has concerned mainly (if not solely) mentally healthy people [11].

Kelley defines chronic self-destructiveness as behaviours involving a generalised tendency to engage in acts that increase the probability of experiencing negative future consequences and/or reduce the probability of attaining positive future ones; perhaps some individuals are constitutionally more responsive to affectively toned sensations than to information-oriented cognition [11–13].

The present work assumes that indirect self-destructiveness is behaviours with negative outcomes intermediated by additional factors, relating behaviour and harm. Indirect self-destructiveness defined in such a way includes not only undertaking, but also abandoning (commission and omission of) actions; it concerns engaging in dangerous and risky situations (i.e. active form) or neglecting one’s own safety or health (i.e. passive form). Furthermore, indirect self-destructiveness is a form of self-destruction of a great distance between an action and outcome. Whereas acute/direct self-destructive behaviour involves conscious and wilful intent to self-inflict painful and injurious acts, sometimes with fatal consequences, chronic/indirect self-destructiveness refers to actions and situations extended over a period of time, with the individual being unaware of or disregarding their long-term harmful effects [14]. The term “indirect” refers not only to the time distance between an action and its harmful consequences, but also to the psychological distance between the kind of behaviour and its psychological and physical consequences [11, 14].

Literature offers studies focusing on particular, isolated manifestations of indirect self-destructiveness in women experiencing domestic violence, e.g. use/abuse of psychoactive substances, poor health maintenance etc. [cf. 15, 16]; there are, however, no studies into indirect self-destructiveness as a generalised behavioural tendency in those women explored in a holistic manner.

The aim of this study was to examine indirect self-destructiveness as a generalised behavioural tendency and its manifestations in women suffering domestic violence.

## Methods

The study is part of a more extensive research project on psycho(patho)logy of women experiencing domestic violence, hence the applied methodology or some other parts of the study may be similar.

### Participants

Two groups of women were studied. The study (criterion, V) group included 52 women aged 30–65 years (mean age: 40.15) using assistance of the Crisis Intervention Centre (CIC) due to experienced domestic violence. Women reported to the CIC on their own initiative or were referred there by an interdisciplinary team for the prevention of domestic violence and all had a “Blue Card”.^1^ The research was carried out by specialists (psychologists) at the start of the intervention, upon informing women about the aim of the research and obtaining their consent to participating in the study. The reference (control, NV) group was well-matched in terms of socio-demographic characteristics and consisted of 150 women not experiencing domestic violence.

### Materials

In order to assess indirect self-destructiveness and its manifestations, the Polish version of the “Chronic Self-Destructiveness Scale” (CS-DS) by Kelley in Suchańska’s adaptation was used. CS-DS comprises several categories of indirectly self-destructive behaviours; the ultimate version is made up of a Likert-type internally consistent set of 52 items with the total obtained score indicating the intensity of indirect self-destructiveness. The research tool encompasses the following categories: Transgression and Risk (A1), Poor Health Maintenance (A2), Personal and Social Neglects (A3), Lack of Planfulness (A4), and Helplessness and Passiveness in the face of problems/difficulties (A5), the scores for which sum up to one global score for indirect self-destructiveness. Both the original scale and its Polish adaptation are characterised by high reliability and validity [12, 14].

## Statistical Analysis

The statistical analysis of received scores applied descriptive and statistical inference methods. In order to describe mean values for quantitative traits, arithmetic means (M) were calculated, while the standard deviation (SD) was assumed to be the dispersion measure. The conformity of quantitative traits’ distributions with the normal distribution was assessed using the Shapiro-Wilk test. Owing to the lack of conformity of dependent variables’ distributions with the normal distribution, the statistical processing of received results applied non-parametric statistics: the Mann-Whitney “U” test to examine inter-group differences and the Kendall’s “tau” (*τ*) correlation coefficient to explore relationships between the studied variables. To examine the structure of relationships between variables and possibly reduce the number of variables, exploratory factor analysis was performed employing the principal component analysis method and normalised varimax rotation; correlation analysis and factor analysis were performed separately for the criterion (V) group and the reference (control, NV) group in order to examine the structure of indirect self-destructiveness for each group. For all the analyses, the maximum acceptable type I error was assumed at α = 0.05. Asymptotic two-sided test probability p was calculated and *p* ≤ 0.05 was considered statistically significant. The statistical analyses were performed by means of the *Statistica PL 13.0* statistical package [17].

## Results

Table 1 shows socio-demographic data of the studied groups; there were no differences in socio-demographic variables because, as mentioned in the Participants section, the reference group was well-matched to the study group in terms of those characteristics.

**Table 1:** Socio-demographic characteristics of studied groups

Variable		Violence		No violence	
		N	%	N	%
Age	M± SD	39.46 ± 8.91		38.17 ± 7.80	
	Range	21–65		22–65	
Marital status	Single	8	15.38	23	15.33
	Non-formalised relationship	3	5.77	9	6.00
	Married	26	50.00	75	50.00
	Divorced	15	28.85	43	28.66
Education	Primary	7	13.46	20	13.33
	Vocational	12	23.08	35	23.33
	Secondary	12	23.08	35	23.33
	Higher	21	40.38	60	40.00

**Fig. 1:**
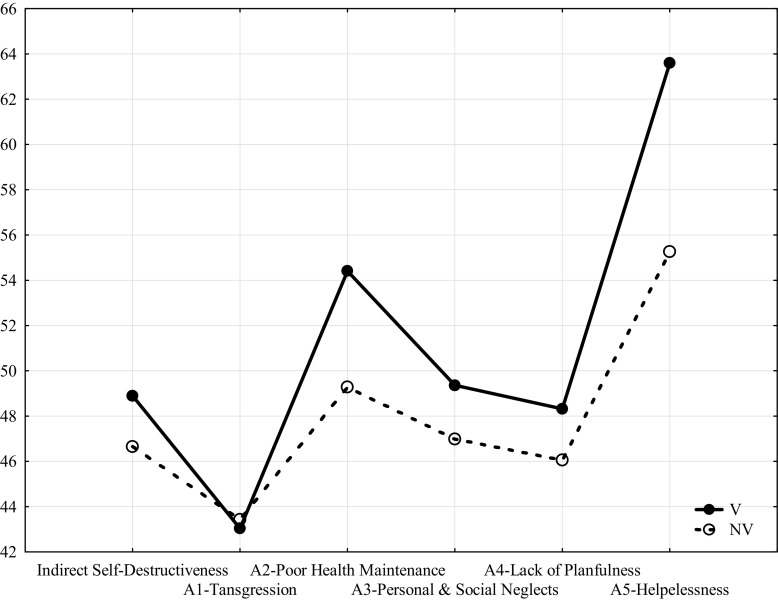
Comparisons of CS-DS scores of women experiencing (V) and not experiencing (NV) domestic violence

Table 2 and Fig. 1 show that women suffering domestic violence (V) obtained significantly higher scores than women in the general population not experiencing domestic violence (NV) for both the general index and a majority of CS-DS subscales. The only exception was the A1 (Transgression and Risk) subscale for which they achieved a somewhat lower score but without statistical significance.

**Table 2:** Comparisons of CS-DS scores of women experiencing (V) and not experiencing (NV) domestic violence

Variables	V group		NV group		U	Significance
	M	SD	M	SD		p
Indirect self-destructiveness	127.135	16.796	121.311	17.201	1830.5	0.03
A1-Transgression and risk	38.731	7.808	39.089	8.068	2308.5	ns.
A2-Poor health maintenance	29.923	5.887	27.100	6.154	1568.5	0.003
A3-Personal & social neglects	29.615	3.941	27.189	5.496	1823.5	0.03
A4-Lack of planfulness	19.327	4.514	17.422	4.499	2101.0	0.05
A5-Helplessness	9.538	1.743	6.630	1.98	1519.5	0.0005

**Fig. 2:**
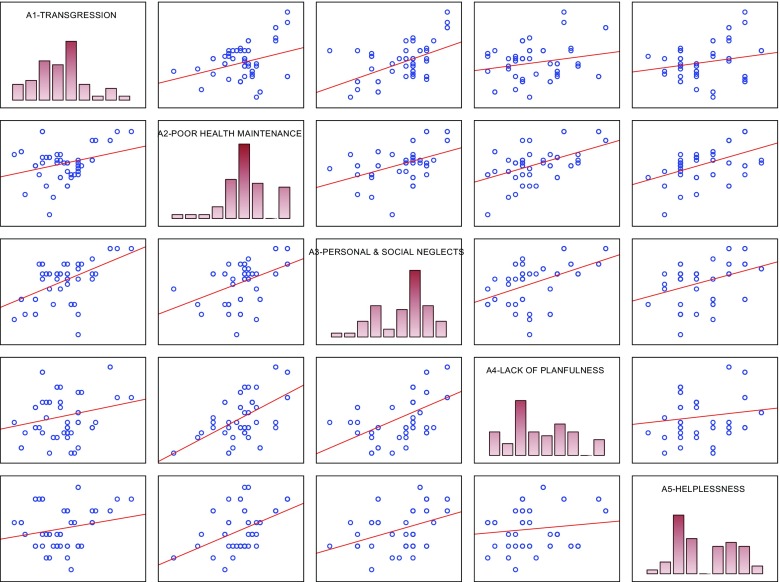
Scatterplot matrix of women experiencing domestic violence (V) scores in the CS-DS subscales

**Fig. 3:**
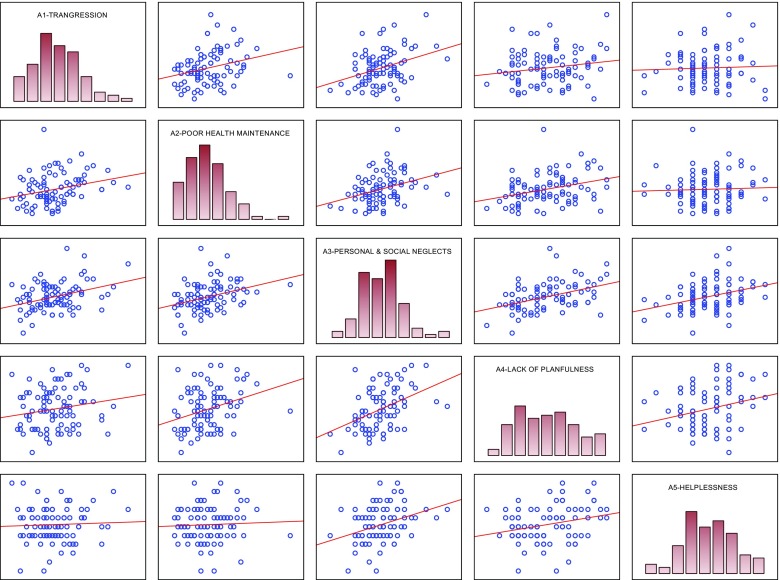
Scatterplot matrix of women not experiencing domestic violence (NV) scores in the CS-DS subscales

In order to examine internal relationships between particular CS-DS subscales, correlations of the subscales were analysed separately for women experiencing (V; Table 3, Fig. 2) and not experiencing domestic violence (NV; Table 4, Fig. 3). Many statistically significant correlation coefficients were found between particular CS-DS subscales for both the groups. It should be noted that the coefficients in the V group were usually higher than in the NV group: statistically significant coefficients ranged from 0.314 to 0.535 in the V group, and from 0.265 to 0.420 in the NV group. Moreover, two differences were observed: In the V group, the A2 (Poor Health Maintenance) subscale was significantly correlated with the A5 (Helplessness) subscale, which did not occur in the NV group; in turn, in the NV group, a significant correlation was found between the A4 (Lack of Planfulness) subscale and the A5 (Helplessness) subscale, which was not observed in the V group.

**Table 3:** Correlation coefficients between CS-DS subscales scores in the group of women experiencing domestic violence (V)

Variables	A1-Transgression	A2-Poor health maintenance	A3-Personal & social neglects	A4-Lack of planfulness	A5-Helplessness
A1-Transgression		0.314 p: 0.02	0.524 *p* < 0.000	0.226 ns.	0.209 ns.
A2-Poor health maintenance			0.444 p: 0.001	0.535 p < 0.000	0.480 p < 0.000
A3-Personal & social neglects				0.516 *p* < 0.000	0.382 p: 0.005
A4-Lack of planfulness					0.139 ns.
A5-Helplessness

**Table 4:** Correlation coefficients between CS-DS subscales scores in the group of women not experiencing domestic violence (NV)

Variables	A1-Transgression	A2-Poor health maintenance	A3-Personal & social neglects	A4-Lack of planfulness	A5-Helplessness
A1-Transgression		0.279 p: 0.008	0.352 p: 0.001	0.181 ns.	0.044 ns.
A2-Poor health maintenance			0.344 p: 0.001	0.326 p: 0.02	0.050 ns.
A3-Personal & social neglects				0.420 p: 0.000	0.347 p: 0.01
A4-Lack of planfulness					0.265 p: 0.01
A5-Helplessness

In order to explore the internal structure of indirect self-destructiveness, factor analysis was performed, employing the principal component analysis method and normalised varimax rotation, for each group separately (Tables 5 and 6 respectively). Results of the analyses differed too. In the V group, only one factor was isolated combining all the CS-DS subscales, the highest loading occurring for the A3 (Personal and Social Neglects) and A2 (Poor Health Maintenance) subscales. That was different in the NV group, in which the factor analysis revealed two factors; for one factor, the highest factor loading occurred for A5 (Helplessness), while for the other – for A2 (Poor Health Maintenance).

**Table 5:** Factor analysis of results obtained by women experiencing domestic violence (V) in the Chronic Self-Destructiveness Scale (CS-DS)

Factors / Variables	Factor loadings	Variance explained
Factor I		
A3-Personal & social neglects	−0.823	50.81%
A2-Poor health maintenance	−0.793	
A4-Lack of planfulness	−0.697	
A1-Transgression & risk	−0.625	
A5-Helplessness	−0.599	
		Total V. Ex.: 50.81%

**Table 6:** Factor analysis of results obtained by women not experiencing domestic violence in the Chronic Self-Destructiveness Scale (CS-DS)

Factors / Variables	Factor loadings	Variance explained
Factor I		40.85%
A5-Helplessness, passiveness	0.841	
A3-Personal and social neglects	0.654	
A4-Lack of planfulness	0.643	
Factor II		20.77%
A2-Poor health maintenance	0.759	
A1-Transgression and risk	0.734	
		Total V. Ex.: 61.62%

## Discussion

While discussing the results, it will be difficult to refer to results of other research in that area because the authors of this study have not found studies dedicated to the issue of interest in available literature. As already mentioned, there are studies solely into particular, isolated manifestations of indirect self-destructiveness in women suffering domestic violence.

It can be inferred from the results received in this study that the intensity of indirect self-destructiveness as a generalised behavioural tendency as well as of most its categories was higher in women experiencing domestic violence. Therefore, it can be assumed that they more frequently and/or intensely display tendencies and behaviours that, although convenient or pleasant at the time, might prove (physically or psychologically) harmful in the long run. Higher indirect self-destructiveness may be yet another aspect and expression of suffering; it may also be an expression of their worse psychological functioning and adaptation. That is an important issue insofar as indirect self-destructiveness is a predictor of direct self-destructiveness, i.e. attempted and committed suicides [18].

The Poor Health Maintenance (A2) category comprises, among others, disregarding physician’s instructions and recommendations as to coping with specific complaints and failure to take actions related to disease prevention, which may ultimately contribute to the worsening of symptoms and signs or even death. Poor Health Maintenance manifestations also include premature discontinuation of treatment, tendency to forget about appointments or procedures, as well as irregular taking of medications or giving that up completely, which is prominent in men. Women experiencing domestic violence neglect their health more, even though women in the general population find it more difficult to avoid contact with physicians, irrespective of their condition, as, for instance, many contraceptives are available only if prescribed, women are more “accustomed” to using health care and more “trained” in that if only due to their essential regular gynaecological check-ups, and more frequently and willingly seek help in the case of health, life, and/or psychological problems [19–23]. Experienced violence makes women neglect many of their matters (as we are going to see further), including their (physical and mental) health, which has already been undermined [cf. 1, 2, 10].

The Personal and Social Neglects (A3) scale assesses neglecting matters of various importance – from trivial ones to those posing an immediate threat to health or even life. Such behaviours of the subject may result in failures or even disasters in one’s life, whose causes the subject may not be aware of. That means that women suffering domestic violence more frequently experience personal and social failures due to abandoning actions that might improve their personal and social situation or their interpersonal relations. An example may be the so called series of misfortunes, i.e. such a manner of acting that decreases the probability of succeeding in one’s actions according to the concept of cognitive dissonance: when experiencing failures, the subject seeks further failures in order not to face a cognitive dissonance situation that might result from achieving success. That particularly dramatic way of regulating one’s expectations by means of the so called strategic failures proves willingness to bear high psychological costs for the sake of a sense of safeness [11, 14]. They may neglect their matters in their willingness to meet the needs of the perpetrator, whose desires have to always take the top priority. Focusing on the perpetrator is likely not to leave space for thinking about themselves, also in the context of taking care of their own health and safety. Furthermore, experienced anxiety associated with dependence on the perpetrator and, in consequence, unpredictability of the setting in which the individual suffering violence functions, may result in abandoning behaviours conducive to safety, development and health.

Lack of Planfulness (A4) is often connected with tendencies to forget about or ignore matters that are significant and important at a certain point in life, and to be careless in everyday life. That may be associated with negative events, apparently unconnected with the subject’s actions, but may directly contribute to endangering the individual’s health or life [11, 14]. Planfulness assumes some relative stability and predictability, which is difficult to achieve in coexistence with the perpetrator: there is most commonly no telling what and when may arouse his anger and aggression, leading to the use of violence.

Higher scores for the Helplessness and Passiveness (A5) index may prove lacking motivation to take specific actions or abandoning them completely when such actions might protect the individual from danger or contribute to ending the suffering of others. That may often contribute to behaviours connected with avoiding or abandoning actions in situations in life that require involvement or taking specific steps aimed at resolving existing problems [11, 14]. Attention ought to be drawn to results of other studies revealing a relationship between indirect self-destructiveness and a sense of impotence and hopelessness [24]. Frequently experienced violence causes a sense of helplessness and impotence in individuals who suffer violence and often do not see a chance of stopping that or extricating themselves from the very unfavourable situation. The helplessness and impotence is also often intentionally created, heightened, maintained and strengthened by the perpetrator who thereby, on the one hand, ensnares his victim (whom he can further abuse) and, on the other hand, ensures his impunity.

A typical, or even textbook, example of indirectly self-destructive behaviours is Transgression and Risk (A1), which may include any behaviours that arouse risky excitation and thrills, and enable an increased adrenaline level in the human body. Interestingly, there were no differences and even a somewhat lower intensity of Transgression and Risk in women experiencing domestic violence. In order to avoid drawing unjustified (due to the lack of statistical significance) conclusions, certain hypotheses for further research may solely be put forward. The observed phenomenon may be an expression of refraining from any kind of risk and overstepping any boundaries, if only in the adaptive aspect of transgression occurring in women [cf. 25], and perhaps from any kind of action for fear of punishment. On the other hand, those women may try to ensure that everything is all right and strive to be “well-behaved” not to arouse the torturer’s anger and aggression. They do not look for thrill, which is characteristic of that type of indirect self-destructiveness, since experienced violence provides them with more than enough of that.

It stems from the above that women suffering domestic violence displayed a more intense passive than active form of indirect self-destructiveness.

As mentioned above, correlation coefficients between CS-DS subscales were higher in the V group than in the NV group, which may indicate that tendencies and categories of indirectly self-destructive behaviours in women suffering domestic violence are more closely connected with one another, and the internal coherence of indirect self-destructiveness in those women might also be higher.

The relationship between Poor Health Maintenance and Helplessness (occurring solely in women suffering domestic violence in contrast to the NV group) may mean that the sense of helplessness resulting from experienced violence may condition poor health maintenance in those women; anyway, as we could see above, women suffering domestic violence neglected to a greater extent more of their matters, including their health.

The lack of correlation between lack of planfulness and helplessness, occurring in the NV group, may mean that lack of planfulness in women experiencing domestic violence is not associated with helplessness. It is quite possible that those women are constantly in the stand-by mode not to provoke the perpetrator’s aggression.

The fact that the factor analysis of women experiencing domestic violence results allowed isolating only one factor may reflect the lack of indirect self-destructiveness internal diversity and uniformity/homogeneity of its structure in those women as opposed to women not suffering domestic violence in whom the internal structure of indirect self-destructiveness was more diversified. Moreover, the fact that the factor analysis produced two factors for NV women may suggest higher internal differentiation and psychological complexity; it may also be assumed that the essence and structure of indirect self-destructiveness differ between women experiencing and not experiencing domestic violence.

The highest share in the structure of indirect self-destructiveness in women suffering domestic violence was that of categories belonging to its passive form, i.e. neglects: social and personal neglects and poor health maintenance, which as a matter of fact was also established in prior analyses.

## Conclusions

Indirect self-destructiveness as a generalised behavioural tendency as well as most categories of indirectly self-destructive behaviours were more intense in women experiencing domestic violence. Moreover, particular categories of indirectly self-destructive behaviours were more closely connected with one another in them, while the internal structure of their indirect self-destructiveness was uniform/homogenous. Stronger indirectly self-destructive tendencies in those women may arise from the experienced domestic violence and be yet another aspect and expression of their suffering. It seems advisable to take into consideration also those aspects of psychological and social functioning in therapeutic work, psychological help and psychosocial actions targeted at women suffering domestic violence.

### Limitations

The (V) sample size may be a possible limitation.

## Footnotes

1 “Blue Card” is an important part of the Polish system of intervention strategies against domestic violence existing in Poland since 1998 and is filled at an intervention site in the presence of the perpetrator.
